# Room Temperature Dehydrogenation of Gaseous Methanol over Polycrystalline Gold Triggered and Traced by Oxygen K-edge X-rays

**DOI:** 10.1021/acs.jpcc.4c06870

**Published:** 2025-01-28

**Authors:** Annette Pietzsch, Johannes Niskanen, Vinicius Vaz da Cruz, Sebastian Eckert, Mattis Fondell, Raphael M. Jay, Xingye Lu, Daniel McNally, Thorsten Schmitt, Alexander Föhlisch

**Affiliations:** † Institute Methods and Instrumentation for Synchrotron Radiation Research, Helmholtz Center Berlin for Materials and Energy, Albert-Einstein-Strasse 15, 12489 Berlin, Germany; ‡ Institute of Physics and Astronomy, University of Potsdam, Karl-Liebknecht-Str. 24-25 , 14476 Potsdam, Germany; § Photon Science Division, Swiss Light Source, Paul Scherrer Institut, CH-5232 Villigen PSI, Switzerland

## Abstract

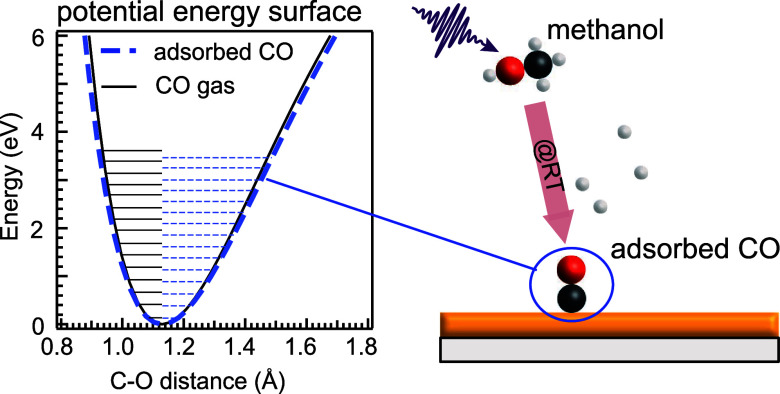

The room temperature conversion of gaseous methanol to carbon monoxide and hydrogen on a polycrystalline Au film at ambient pressure has been triggered and characterized by oxygen K-edge excitation and vibrationally resolved resonant inelastic X-ray scattering. The rate-limiting first methanol dehydrogenation step is driven by ultrafast O–H dissociation and deprotonation of O K-edge excited CH_3_OH. The Au surface further dehydrogenates the CH_3_O^+^ photoradical created by X-rays via electron transfer from the Au surface. With vibrationally resolved resonant inelastic X-ray scattering, we trace the CO molecular potential energy surface along the C–O coordinate. The CO bond softens, and the C–O stretch frequency changes from 2250 to 2065 cm^–1^ at a CO chemisorption energy of 38–58 kJ/mol. This constitutes weak chemisorption as compared to the transition metals but also stronger bonding than the physisorbed CO species on single-crystal Au surfaces. In liquid methanol, the recombination of the CH_3_O^+^ photoradical created by X-rays with protons quenches this conversion.

## Introduction

Surface catalytic (de)hydrogenation involving methanol (CH_3_OH) and carbon monoxide (CO) constitutes an important aspect in itself and as a side pathway within the water–gas shift reaction of hydrogen generation. In catalysis, the first step of methanol dehydrogenation is rate-limiting.^1^ It can be carried out over various metal surfaces at elevated temperatures, where intermediates and the heats of adsorption strongly influence reaction pathways. Here, the interfacial region at the metal surface modulates the electrocatalytic properties and defines the probability of certain catalytic products. In particular, weakly chemisorbed CO is crucial to avoid catalytic poisoning through surface passivation,^2^ making the heat of adsorption of the methanol decomposition products an important quantity. Across the transition and noble metal series, product selectivity varies:^3^ In noble metals, Cu surfaces allow for a range of molecular species, whereas Au is highly selectively linking CH_3_OH to CO and H_2_.

The conversion of methanol on gold takes place at temperatures around 160°, which can be lowered to 60 °C by adding iron or aluminum oxide to the gold catalyst.^4^ The metal oxides take part in the reaction pathway by offering surface lattice oxygen atoms as anion vacancies and additional oxygen adsorption sites, thus lowering the temperature barrier. Carbon monoxide’s ability to allow for surface chemical bonds of varied interaction strength makes it a central catalytic moiety,^5^ spanning from weak physisorption to strong chemisorption.^6^ This depends on the detailed electronic structure of the substrate, the local bonding site, and adsorbate–adsorbate interactions. Gold allows both physiorbed and chemisorbed species. The heat of adsorption on low-index surfaces is very small (around 10 kcal/mol^7^), but stronger interactions have been observed on Au nanoparticles with stepped surfaces.^4^ The strength of CO adsorption is reflected in the C–O vibrational stretch frequency. Since bond order is conserved, a stronger CO-metal surface bond weakens the intramolecular C–O bond. Thus, this softening of the C–O stretch is an indicator of adsorption strength.

In this work, we replace the first rate-limiting thermal dehydrogenation step of methanol by X-ray-induced deprotonation occurring at room temperature.^8^ Then, we follow the charge transfer steps on the Au surface toward CO and H_2_ by high-resolution inelastic X-ray scattering (RIXS) at the oxygen K-edge. We monitor the accumulation of adsorbed CO over time, probing the valence electronic structure of both methanol and CO, and describe with first-principles computations the species along the proposed reaction pathway. In particular, electrons from the Au surface transfer to the CH_3_O^+^ photoradicals created by X-rays, which drives the conversion of methanol to hydrogen and CO adsorbed on the Au surface. We determine the CO/Au heat of adsorption as 38–58 kJ/mol and determine the potential energy surface of this weakly chemisorbed CO/Au species.

## Experiment

A 10 nm thick layer of Au was deposited on a Si_3_N_4_ membrane (150 nm Si_3_N_4_ purchased from Silson) by vapor deposition at room temperature, corresponding to roughly 75 monolayers. The Si_3_N_4_/Au membranes were mounted in a gas flow cell with the Au layer pointing toward the gas. Liquid methanol was heated to 40 °C, and the vapor was collected and injected into the flow cell at a pressure of 150 mbar. RIXS spectra with vibrational resolution were measured at an incidence angle of 45° and a total scattering angle of 90° with linear vertical polarization of the X-rays. The spectra were measured using the SAXES spectrometer^9^ at the ADRESS beamline^10^ at the Swiss Light Source of the Paul Scherrer Institute. The excitation energy was set to 534.0 eV (covering both the methanol and the CO π* resonance) to track the oxidation of methanol and the creation of CO on the Au film. The overall resolution was 45 meV. All RIXS spectra have been normalized to the measurement time.

The RIXS spectra detect electronic excitations as well as the vibrational progression of the methanol gas. In a series of subsequent short spectra measured at the same spot, we tracked the conversion of methanol to CO at the Au surface. RIXS is uniquely powerful to reach site-selective electronic structure information in combination with ground state potential energy surface mapping via vibrational progression. In this, the direct Franck–Condon occupation allows us to trace potential energy surfaces from equilibrium to strongly distorted geometric species via the structural strong push along normal mode coordinates during the transient core-excited state. This is an essential difference to vibrational spectroscopy with IR or Raman approaches, where multistep excitation of vibrationally highly excited states occur. RIXS is also element and chemical state-selective, separating, for our case here, the methanol and CO signatures fully. Assuming a C–O Morse potential (as is reasonable for free or only weakly interacting molecules), we then derive the ground state potential energy surface (PES) of the adsorbed CO molecules. The PES of gas-phase CO is known from previously published RIXS data.^11^ The methanol fragment energies were calculated at the DFT/B3LYP level of theory by using the def2-TZVP(-f) basis set and the D3BJ dispersion correction. The energy of the core-excited species was taken from the experimental value. All calculations used the ORCA package.^12^

## Results and Discussion

**Figure 1:**
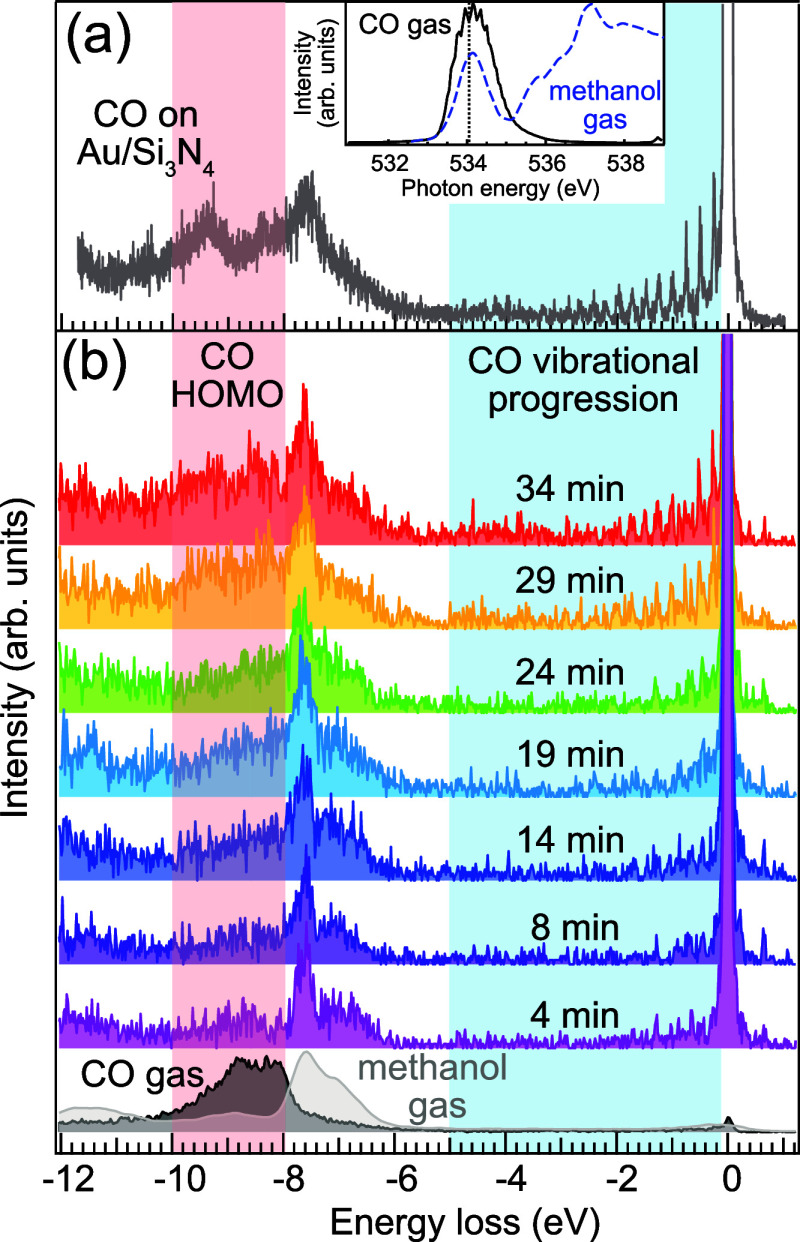
Conversion of methanol to CO probed by O K-edge RIXS: (a) RIXS spectrum excited at 534.1 eV of CO adsorbed on the Au/Si_3_N_4_ surface. The inset shows the O K-edge NEXAFS spectra of gas-phase CO (black) and gas-phase methanol (blue, data from ref (13)) with the RIXS excitation energy markes with a dashed line. (b) Temporal evolution of O K-edge RIXS spectra of gas-phase methanol at a Au/Si_3_N_4_ surface excited at the same energy. The spectra show the slow appearance of an additional peak around −9 eV energy loss starting at roughly 14 min exposure. The feature’s energy coincides with that of the CO gas HOMO (black curve at the bottom of panel (b), data from ref (11)), whereas the energy region −6 to −8 eV loss is dominated by the methanol gas signal (gray curve in the bottom of panel (b), data from^14^). In the range of −0.1 to −5 eV energy loss, the developing vibrational progression is observed, its vibrational energies matching those of the CO gas.

In Figure 1, we show the experimental K-edge RIXS data of methanol converting into CO on Au gradually. The near edge X-ray absorption spectrum (NEXAFS) at the oxygen K-edge of CO and gas-phase methanol are shown in the inset of Figure 1a (gas-phase CO from ref (13)). The main resonance of CO is energetically very close to that of methanol; thus, RIXS spectra excited at 534.1 eV are sensitive to both moieties. The different contributions can be separated via the distinct shapes of the X-ray emission features.

RIXS of the methanol gas at the Au surface with 534.1 eV excitation energy thus gives a picture of the methanol and CO population. Over time, we observe that the distinct methanol spectral fingerprint becomes broader, and a new feature in the range of 8–10 eV energy loss (corresponding to the energy of the CO emission features) starts to emerge. At the same time, for low-energy losses up to 5 eV, a vibrational progression with the first overtone frequency of 2065 cm^–1^ starts to develop. This indicates that with increasing irradiation time, the methanol signal is superposed with the RIXS signal originating from CO. The developing vibrational progression is very close in frequency to that of gaseous CO, and the new spectral feature at 8–10 eV energy loss coincides with the most prominent RIXS feature of CO. This is an indicator of the production of CO at the surface.

Figure 1a shows the RIXS spectrum of adsorbed CO at the Au/Si_3_N_4_ surface excited at the π* resonance at 534.1 eV. We find a pronounced vibrational progression in the energy range from 0 to −5 eV energy loss as well as a double-peak structure between −7 and −10 eV energy loss. The adsorbed CO is produced by the photoconversion of methanol gas; in panel (b), we show the evolution of methanol gas decomposition and CO accumulation at the Au surface; all spectra are normalized to the measurement time. The intensity development of the CO and methanol gas contributions shows that the methanol is quickly (in a matter of minutes) converted into CO (see Supporting Information for further information).

**Figure 2:**
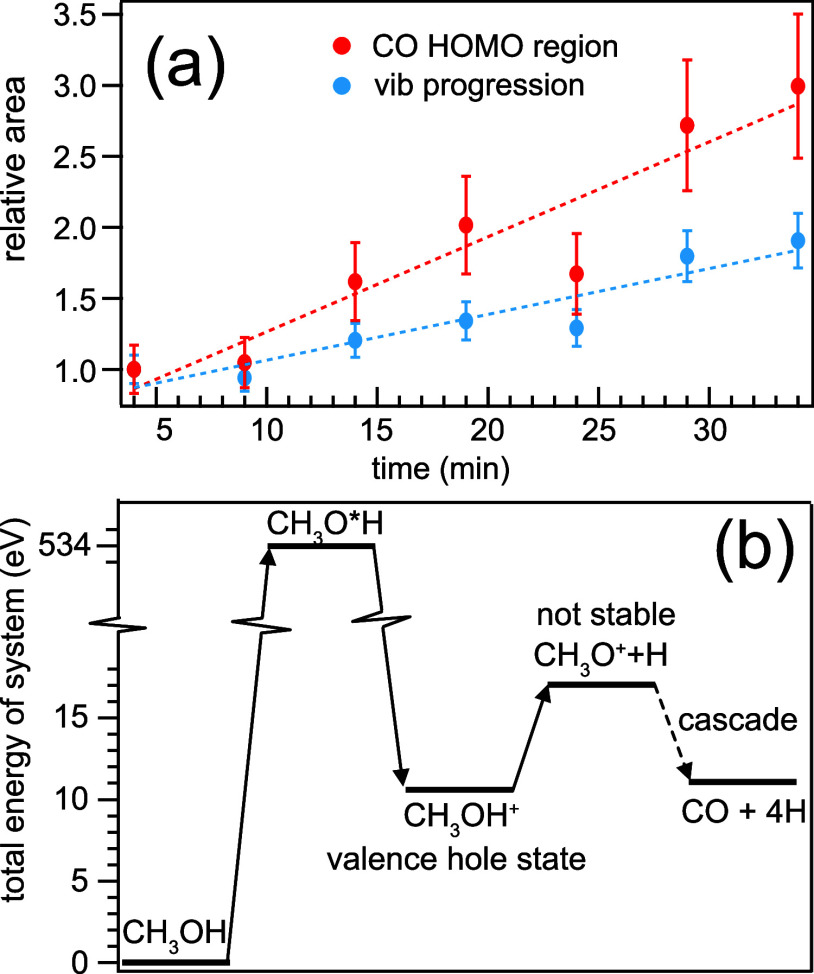
(a) Temporal evolution of the CO contribution to the RIXS spectra collected at the Au/Si_3_N_4_ surface excited at 534.1 eV. The change in area in the range of −8 to −10 eV energy loss (red, CO HOMO region) and −0.1 to −5 eV energy loss (blue, CO vibrational progression) illustrate the linear behavior of the CO accumulation on the Au surface with time. (b) Energetics of the involved species from educt to product based on first-principles computations.

The conversion happens at room temperature and is driven by the X-ray core excitation of methanol and the consecutive electrochemical processes. The reaction pathway with the respective total energies of the methanol system is shown in Figure 2a, with uncertainties corresponding to the respective spectral noise within each spectrum, determined by the equivalent spectral region on the anti-Stokes side (3–8 eV). The directional selectivity of RIXS is uniquely sensitive to the methanol O–H bond elongation, and at the same time, it allows (due to the energetic overlap with the C–O X-ray resonance) to simultaneously detect how the C–O stretch occurs as the reaction builds up the CO product. Resonant excitation at the methanol O K-edge drives the local O–H bond elongation and leads to deprotonation of the methanol molecule from CH_3_OH to CH_3_O^+^ within the core-excited state lifetime.^8^ At a synchrotron, the number of scattering events during the scattering duration is exclusively single-photon events, meaning that the methanol dissociation is triggered by single-photon excitation. The subsequent CH_3_O^+^ photoradical created by X-rays is unstable, and electrons from the Au surface lead to the conversion into CO on the Au surface. Since larger hydrocarbon species as reaction products only occur in significant amounts at Cu surfaces,^2,15^ for Au, the products are selectively CO and H_2_.^3^

At the bottom of Figure 1b, two reference RIXS spectra at 534.1 eV for CO gas (from ref (11)) and methanol gas (from ref (14)) are shown. We clearly see that the spectral contribution from the CO is concentrated in the energy loss range between −10 and −8 eV, whereas methanol gas shows its most prominent RIXS features in the energy range from −6 to −8 eV energy loss. This means that we can distinguish their different spectral contributions.

To follow the temporal evolution of the methanol conversion to CO, we measured a series of consecutive spectra (with 4 min acquisition times each) at 534.1 eV excitation energy (see the gray dashed line in the inset of Figure 1), while monitoring the increase in CO signal. The spectrum after 4 min is still very similar to that of methanol gas; but within 10 min, a broad feature is developing in the range between −8 and −10 eV energy loss, in the region where the CO highest occupied molecular orbital (HOMO) is situated. At the same time, the intensity at low-energy loss is increasing, and a vibrational progression is developing in the region between 0 and −5 eV. The delayed appearance of vibrational progression is key evidence of a reaction taking place and is characteristic to its products. The energy spacing of the vibrational overtones matches that of CO gas very closely, which we will discuss later in greater detail.

To quantify the spectral changes and monitor the CO production and accumulation on the Au surface, we plot the area *A*_rel_ relative to the reference spectrum at 4 min (*A*_rel_ = *A*_spec_/*A*_ref_) in Figure 2a, focusing on the fingerprint regions defined earlier. We find that both the intensity of the electronic states around the CO HOMO and the vibrational progression increase linearly with time, with the energy loss region of the electronic excitations being more sensitive to the CO signal.

In Figure 2b, DFT calculations of the methanol system's total energy during the conversion process are shown. The methanol conversion to CO is triggered by resonant soft X-ray excitation at the O K-edge, driving the deprotonation of the methanol O–H on the time scale of the femtosecond oxygen core hole lifetime, without reaching thermal equilibrium. The product is an unstable CH_3_O^+^ radical that then decays in a cascade to the final decomposition products CO + 4H, with the help of electron donation from the Au surface, sizable enough to lead stepwise to adsorbed CO.

The CO accumulation on the Au happens on a time scale of around 10 min in both fingerprint regions. Since the valence electronic structure of CO is modified upon adsorption due to the rehybridization of the CO π system with the *t*_2g_ states of the metal d-band^6^ (which introduces a systematic error in the comparison), we focus on the CO fingerprint in the C–O stretch vibrational progression. Here, we expect a variation in the C–O bond distance together with shifts in C–O stretching frequency in comparison to the gas-phase molecule, as has been observed depending on the adsorbate system.^16,17^ We see no spectral contribution of intermediates such as the Au-bound methoxy species that have been reported for Pt^18^ and Mo^19^ surfaces.

**Figure 3:**
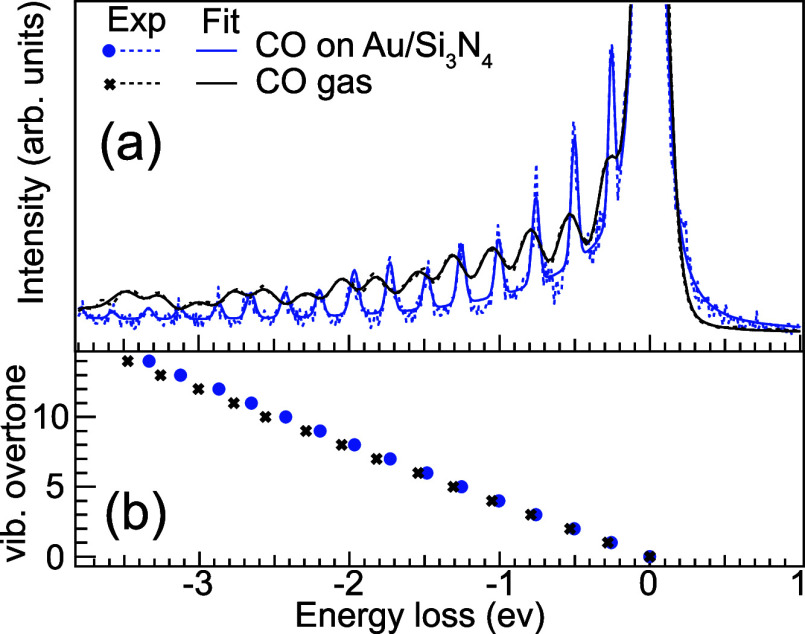
(a) Low-energy vibrational loss region of high-resolution RIXS spectra excited close to the CO π* resonance at 534.0 eV of CO gas (black, data from ref (11)) and CO on Au/Si_3_N_4_ (blue) shown together with a fit of the vibrational progression. (b) Energy of each overtone in the vibrational progressions. We observe an increasing energy difference with overtone number between the gas-phase and adsorbed CO.

In Figure 3, the vibrational progressions from high-resolution RIXS spectra of gas-phase CO (black, from ref (11)) and CO adsorbed on Au/Si_3_N_4_ (blue) are shown in the low-energy loss region. We find the vibrational progression of adsorbed CO to be extremely close to that of CO gas, which is assigned to the fact that the heat of adsorption of CO on Au is small. Nevertheless, we now concentrate on the question whether we can obtain any detailed information on how the CO binds to the surface and how the adsorbed CO differs from a free CO molecule. To achieve this, we use high-resolution RIXS to extract information on the ground state PES of CO, a tool that we have developed over the past 15 years.^20−25^ The site specificity and chemical selectivity of RIXS allow extraction of a cut through the ground state PES at an atomic center of the molecule along a nuclear coordinate; in the case of CO, we investigate the cut along the C–O bond, exciting the oxygen atom at 534.1 eV.

The vibrational progressions have been fitted with a series of Voigt profiles, where the Lorentzian width is defined by the width of the elastic line at 0 eV energy loss and kept constant throughout the fit. The Gaussian width (left free in the fit) represents additional coupling.

Due to different experimental conditions, the total energy resolution differs between the two spectra (the experimental resolution of the CO gas measurements was about 150 meV, whereas it was 50 meV in the methanol gas experiments). However, this does not affect our finding since a free CO molecule shows a single-mode vibrational spectrum; thus, internal vibrational coupling is impossible. Here, we clearly observe that the vibrational overtone energies of the adsorbed CO molecules are always less than those of free molecules, indicating a weakened C–O bond due to the interaction with the Au substrate.

It should be noted that this interpretation implies a dispersed molecular system, which is not always true for adsorbates. In the case of CO on Au, however, the heat of adsorption is so low that only a dilute population of the Au surface is realized. A more extensive adsorption rate would arguably be balanced by desorption due to the repulsive dipole–dipole interaction between two molecules.

**Figure 4:**
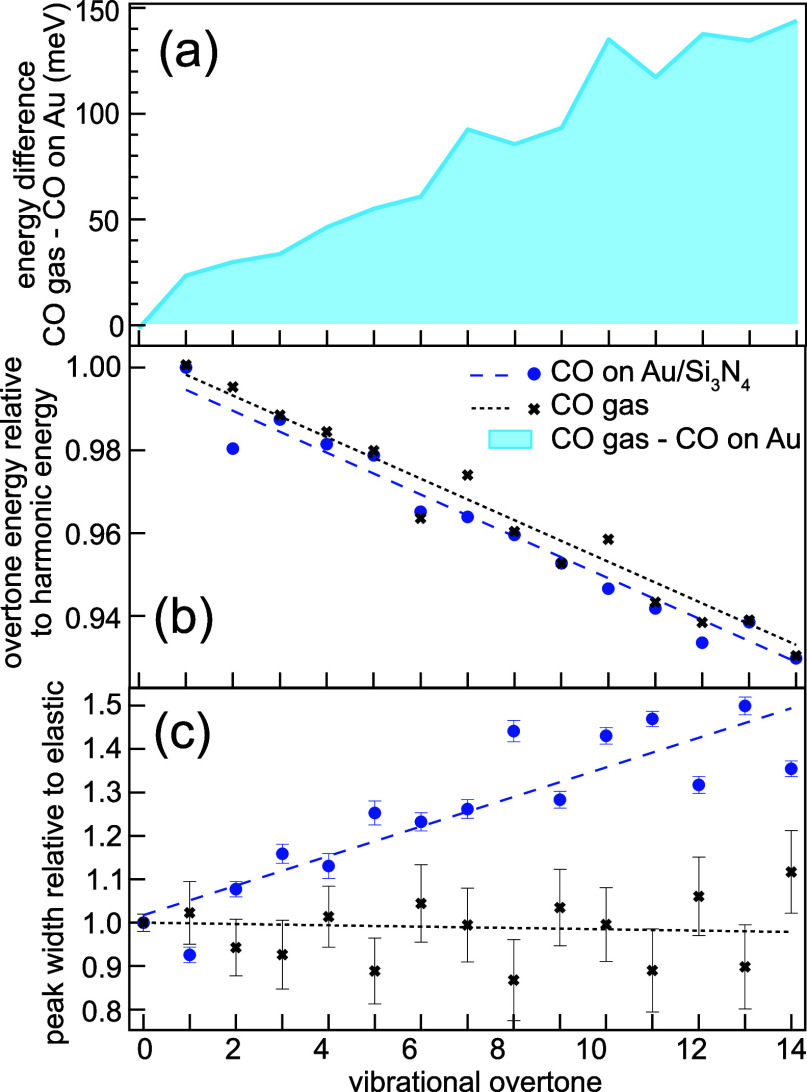
Comparison of CO-stretch vibrational progression characteristics for CO gas (black) and CO adsorbed on Au/Si_3_N_4_ (blue): (a) the energy difference (light blue area) between the overtone energies of CO gas and adsorbed CO shows a linear increase with overtone number. (b) The ratio of overtone energy to harmonic energy is linear with the overtone number, indicating that the PES shape is Morse-like.^25^ (c) The Gaussian peak width relative to the elastic line is constant for the free molecule in CO gas, whereas the peak width of the adsorbed CO is increasing with overtone number, a signature of the molecules coupling to the substrate. The dashed and dotted lines are linear fits to the data.

To quantify the differences in the vibrational progressions, we investigate the overtone energies and Gaussian peak widths as a function of overtone number in Figure 4. In panel (a), the energy difference (light blue area) between the overtone energies of gas-phase CO and CO adsorbed on Au (black crosses and blue dots in Figure 3b) is shown. We find a linear dependence on the overtone number, where the overtone energy of adsorbed CO is always weaker than that of free CO in the observed energy range.

Complementary to IR and Raman measurements, we can with vibrationally resolved RIXS access a large number of vibrational overtones in the electronic ground state of the molecule and with that gain information on the molecular system further away from equilibrium. The energy spacing of the vibrational overtones is defined by the shape of the molecular PES. This means that we can use our measured vibrational progression to extract the PES along the C–O bond for both free and adsorbed C–O.

To verify the PES shape, in Figure 4b, we plot the overtone energies relative to the respective energies in a harmonic potential defined by the first overtone energy. This will give a constant line for a harmonic PES and a linear dependence on the overtone for a Morse-like PES.^25^ Here, we find similar linear slopes for free and adsorbed CO that indicate clear Morse shapes of the two PES.

The widths of the vibrational overtones contain information about molecular interaction. An unperturbed molecule shows a constant overtone width throughout the spectrum, whereas broadening for higher overtones indicates coupling to the environment. For both free and adsorbed CO, in Figure 4c, we plot the overtone Gaussian peak width relative to the Gaussian width of the elastic line. The latter one is dominated by the experimental resolution; taking this as a reference, we are sensitive to broadening effects due to environmental effects such as mode coupling or overlapping vibrational profiles from different structures.

For gas-phase CO, the relative width stays constant and close to unity, as it is expected for free, noninteracting molecules. The single-mode vibration of free CO guarantees that only one vibrational frequency contributes to the spectrum. For CO adsorbed on Au/Si_3_N_4_, however, we find a linearly increasing broadening of the vibrational overtones with the overtone number. This is an effect of the bonding to the substrate, creating a manifold of CO molecules with slightly different vibrational frequencies and coupling to soft vibrational modes, which is absent in the isolated molecule.

**Figure 5:**
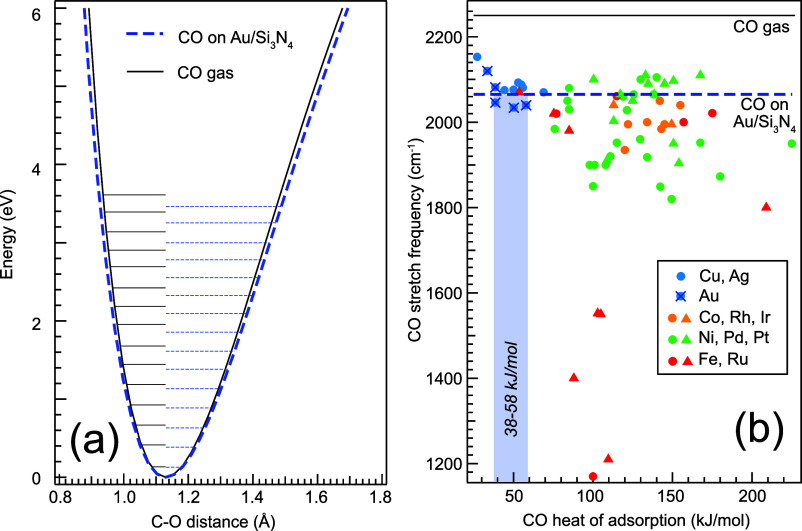
Bond softening and heat of adsorption: (a) comparison of the potential energy surfaces along the C–O bond of gas-phase CO and CO adsorbed on Au/Si_3_N_4_ from vibrationally resolved oxygen K-edge RIXS. (blue: adsorbed CO, black: gas-phase CO from previous work^11^); the horizontal lines indicate the measured vibrational overtone energies. (b) C–O stretch frequency of CO adsorbed on different transition metal surfaces plotted against the respective heat of adsorption (circles) or activation energy of desorption (triangles). The colors denote different electronic configurations of the metals: blue circles are associated with the closed shell d^10^ (half-)noble group 11 metals Cu, Ag, and Au; green circles are associated with the group 10 metals Ni, Pd, and Pt; yellow circles are associated with the group 9 metals Co, Rh, and Ir; and red circles are associated with the group 8 metals Fe and Ru. The data is taken from refs (26−28). The C–O stretching frequencies of free (2250 cm^–1^) and adsorbed CO measured in this work (2065 cm^–1^) are marked by horizontal lines. Comparing the C–O stretch frequency obtained from our measurements to those for CO on noble metals and, in particular, CO on Au, we can estimate the corresponding heat of adsorption for CO on Au/Si_3_N_4_ to be between 38 and 58 kJ/mol (light blue box).

Extracting now the full PES for both free and adsorbed CO with the validated Morse ansatz leads to the PES shown in Figure 5a (black and blue curves, respectively). From the RIXS vibrational progression, the PES is extracted relative to the C–O equilibrium distance; here, we assume for both PES the C–O equilibrium distance of free CO of 1.13 Å.^29^ Calculations for CO on different Au surfaces show only small changes in the C–O bond length of up to ±0.01 Å^17^ compared to free CO, making this a valid assumption. Comparing the two PES, we can now study the effects of adsorption on the CO bond. We find that the PES of adsorbed CO is wider than that of the free molecule, quantifying how the adsorption on the Au film weakens the C–O bond.

In order to relate heat of adsorption to C–O stretch frequency, we link in Figure 5b for CO adsorbed on metal surfaces the known experimental C–O stretch frequencies (first overtone) to the heats of adsorption (data taken from refs (26−28)). The values are grouped according to electron configurations of the metals. We observe that CO on noble metals (blue circles in Figure 5b) shows a trend of decreasing C–O stretch frequency with increasing heat of adsorption. Comparing our measured frequency (2065 cm^–1^, blue dashed line in the figure) to the literature values for CO on Au surfaces, we derive the heat of adsorption for CO on Au/Si_3_N_4_ to be between 38 and 58 kJ/mol. This energy range constitutes weak chemisorption compared to the transition metals but also stronger bonding than the physisorbed CO species on single-crystal Au surfaces.

The relatively weak heat of adsorption for CO on noble metals can be explained by significant orbital mixing as attractive covalent interaction of the CO π orbitals with the *t*_2g_ states of the metal d-band and a competing repulsion of the CO σ system with the *e*_g_ states of the metal d-band.^6,17^ We observed the effect of these interactions directly through the broadening of the vibrational overtones in adsorbed CO.

Methanol conversion to CO over polycrystalline Au takes place only for gaseous methanol but not for liquid methanol. Despite the fact that molecular methanol fragmentation by core excitation happens similarly for gas and liquid methanol, the probability for recombination with a proton is dominant in the liquid and nonexistent in the gas phase. This recombination mechanism quenches the further dehydrogenation steps over Au in the liquid phase and has been previous established for X-ray-induced O–H bond breaking in liquid acetic acid.^24^

## Conclusions

The room temperature conversion of gaseous methanol to carbon monoxide and hydrogen on a polycrystalline Au film at ambient pressure has been triggered and characterized by oxygen K-edge excitation and vibrationally resolved resonant inelastic X-ray scattering. The rate-limiting first methanol dehydrogenation step is given by ultrafast O–H dissociation and deprotonation of O K-edge excited CH_3_OH. The Au surface further dehydrogenates the CH_3_O^+^ photoradical created by X-rays via electron transfer from the Au surface. With vibrationally resolved resonant inelastic X-ray scattering, we trace the CO molecular potential energy surface along the C–O coordinate. Comparison of the vibrational progressions measured with high-resolution RIXS for both free and adsorbed CO shows the evidence of the Morse-like C–O PES for both species. The adsorbed CO shows both C–O bond softening and broadening of the higher vibrational overtones due to the adsorbate interaction. The C–O stretch frequency of 2065 cm^–1^ in comparison to that of gas-phase CO (2250 cm^–1^) indicates a heat of adsorption within the range of 38–58 kJ/mol. This energy range constitutes weak chemisorption as compared to the transition metals but also stronger bonding than physisorbed CO species on single-crystal Au surfaces. We also note that only in the gas phase can the CH_3_O^+^ photoradical created by X-rays convert to CO over the Au surface. In liquid methanol, swift recombination of the CH_3_O^+^ photoradical created by X-rays and the proton quenches the conversion into CO over the Au surface.

## Supporting Information Available

Discussion of the temporal evolution of the CO spectral fingerprint in the O K-edge RIXS spectra during the conversion of methanol to CO over a polycrystalline Au surface (PDF)
