# Supplementary material for: Room Temperature Dehydrogenation of Gaseous Methanol over Polycrystalline Gold Triggered and Traced by Oxygen K-edge X-rays
Source: J Phys Chem C Nanomater Interfaces. 2025 Jan 28;129(5):2453–9. doi: 10.1021/acs.jpcc.4c06870 (PMC11808772; doi:10.1021/acs.jpcc.4c06870)
Supplement: Supplementary file 1 — jp4c06870_si_001.pdf [file jp4c06870_si_001.pdf]

Supporting Information: Room Temperature Dehydrogenation of Gaseous Methanol over Polycrystalline Gold Triggered and Traced by Oxygen K-edge X-rays

Annette Pietzsch,[*,†] Johannes Niskanen,[†,§] Vinicius Vaz da Cruz,[†] Sebastian Eckert,[†] Mattis Fondell,[†] Raphael M. Jay,[‡,∥] Xingye Lu,[¶] Daniel McNally,[¶] Thorsten Schmitt,[¶] and Alexander Föhlisch[†,‡]

†*Institute Methods and Instrumentation for Synchrotron Radiation Research, Helmholtz Center Berlin for Materials and Energy, Albert-Einstein-Strasse 15, 12489 Berlin, Germany*

‡*Institute of Physics and Astronomy, University of Potsdam, Karl-Liebknecht-Str. 24-25, 14476 Potsdam, Germany*

¶*Photon Science Division, Swiss Light Source, Paul Scherrer Institut, CH-5232 Villigen PSI, Switzerland*

§*present adress: Department of Physics and Astronomy, University of Turku, 20014 Turun yliopisto, Finland*

∥*present adress: Department of Physics and Astronomy, Uppsala University, Box 516, 751 20 Uppsala, Sweden*

E-mail: annette.pietzsch@helmhholtz-berlin.de

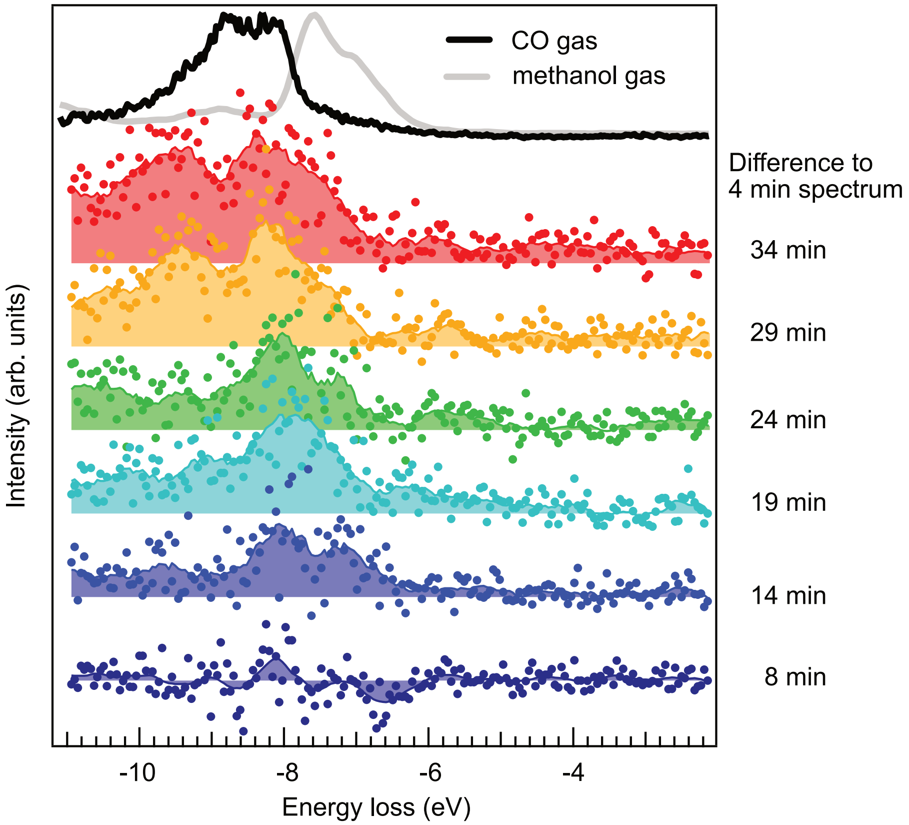

Figure S1: Difference of each single RIXS spectrum in Fig. 1 in the manuscript to the initial spectrum at 4 min. The difference spectra (dots) have been smoothed (filled curves) as a guide to the eye. We find that with increasing time, the signal in the energy region that corresponds to CO gas increases, while the signal in the methanol gas region stays constant or even decreases. The upper part shows the comparison to the RIXS of CO gas and methanol gas.

Supporting Information Available

Temporal evolution of the O K-edge RIXS spectra of methanol gas on Au/Si_3N_4

The intensities of the different constituents in the RIXS spectra of methanol gas at the Au/Si_3N_4 surface are the result of partial pressures and their specific probing depths based on the absorption cross sections. In particular, the low density of the gas phase sample allows the methanol signal to be detected from the whole cell volume behind the membrane.

In contrast, the CO signal is detected from the CO that is only formed on the back of the membrane. That means in early times when the CO coverage of the membrane is still small, we also detect the methanol in the cell that is replenished from a reservoir during measurement, keeping the methanol signal intensity still constant. For a fuller CO layer, the methanol signal then decreases. What we observe in the manuscript in Fig. 1 as an increase in the methanol energy region (-8 to -6eV) is actually a tail of the increasing CO signal that pushes the methanol up.
